# Dr. Edwin Mayo Bangs

**Published:** 1917-02

**Authors:** 


**Dr. Edwin Mayo Bangs,** Boston University 1878, died at his home in Ithaca, Dec. 24.

